# FDG‐PET and Amyloid PET scans: benefits beyond Alzheimer´s Disease. It´s really useful in daily practice? Clinical case review

**DOI:** 10.1002/alz.087859

**Published:** 2025-01-09

**Authors:** Shenda Orrego, Mihaela Sava, Maria Pilar Garcia, María Sagrario Manzano

**Affiliations:** ^1^ Universitary Hospital Infanta Leonor, Madrid, Madrid Spain; ^2^ Psychiatric Department, Madrid Spain; ^3^ Psychiatric Department, Hospital Infanta Leonor, Madrid Spain; ^4^ Head of Nuclear Medicine Department, Universitary Hospital Getafe, Madrid Spain; ^5^ Neurology Department Infanta Leonor Hospital, Madrid Spain

## Abstract

**Background:**

biomarkers are essential in order to make a diagnosis with a high level of accuracy in patients with cognitive and behavior complaints. However, molecular imaging biomarkers not always provide an answer in daily clinical practice.

**Methods:**

retrospective and descriptive study in patients with Amyloid PET (APscans) implemented according to rational use of this technic, between January 2019‐November 2023 in Neurology Department, Infanta Leonor Hospital, Madrid, Spain.

We included: first visit date, type of diagnosis, date, age, sex, neuropsychiatric symptoms, doctor requesting the diagnosis, CT/MRI scans (atrophy or vascular signs), NPS values (GDS),18F‐FDG PET scans (divided into normal, AD pattern, not AD pattern, indeterminate). The study was approved by the Research Committee of ILH. Statistical analysis was made with Dataset and SPSS 22.0.

**Results:**

total of 51 patients (54.9%females) were included. Mean of age 61.058, SD:8.329

The mean of months till diagnosis was 10.82, SD:10.133. 74.5% cases were GDS 3 in the first visit.

Positive APScans:39.22%, negative APScans:50.98%, not made yet:1.96%, not made:7.84%

In the negative APScans only 26.92% could obtain a diagnosis with a high level of accuracy (Lewy body dementia 11.54%; FTD. 15.38%). But in 23.08% the results were not conclusive and the patients remains without a diagnosis in the follow up.

In FDG‐PET‐scans we observed an impairment of glucose metabolism in 72.55% of patients. 58.82% FDG‐PET‐scans have a not AD pattern, being indeterminate in 73.33%.

49.02% patients have history of depression, 3.92% psychosis, sleep disorders 1.96%, anxiety 3.92%, suicidal ideation 1.96%. Positive APScans has depression in 50% of cases vs 57.69% of negatives(p>0.05). Psychosis was present in 10% of positive APScans vs 0% of negatives(p>0.05). These symptoms did not predict the positive or negative results in APScans

In structural neuroimaging we observed 55% of patients with atrophy in MRI scans. A vascular pathology in MRI (lacunar strokes or leukoaraiosis):56.86%. Vascular pathology in structural neuroimaging predicted the higher probability to have a negative APScan (57.69%). Probably the small size could impact in all these results.

**Conclusion:**

In patients with cognitive and behaviour complaints, biomarkers are essential to make a diagnosis. However, when an APScan is negative and FDG‐PET‐scan indeterminate, diagnostic uncertainty, in real life, remains a serious unresolved issue

